# Progesterone inhibition of oxytocin signaling in endometrium

**DOI:** 10.3389/fnins.2013.00138

**Published:** 2013-08-07

**Authors:** Cecily V. Bishop

**Affiliations:** Division of Reproductive and Developmental Sciences, Oregon National Primate Research Center, Oregon Health & Science UniversityBeaverton, OR, USA

**Keywords:** oxytocin, oxytocin receptor, progesterone, non-genomic steroid hormone action, non-classical steroid hormone action

## Abstract

Expression of the oxytocin receptor (OXTR) in the endometrium of ruminant species is regulated by the ovarian steroids progesterone (P) and estradiol (E). Near the end of the estrous cycle, long-term exposure of endometrial epithelial cells to P results in loss of genomic P receptors (PGRs), leading to an increase in E receptors (ERs). Genomic regulation of the OXTR is mediated via suppression of ER signaling by P. Upon OT binding at the plasma membrane of endometrial cells, a signaling cascade is generated stimulating release of prostaglandin F_2α_ (PGF_2α_). Transport of PGF_2α_ to the ovary results in release of OT by luteal cells in a positive feedback loop leading to luteal regression. This signaling cascade can be rapidly blocked by exposing endometrial cells to physiologic levels of P. This mini review will focus on the mechanisms by which P may act to block OXTR signaling and the luteolytic cascade in the ruminant endometrium, with special focus on both non-genomic signaling pathways and non-receptor actions of P at the level of the plasma membrane. While this review focuses on ruminant species, non-classical blockage of OXTR signaling may be important for fertility in women.

## Introduction

In mammals, signaling by the neuropeptide oxytocin (OT) via receptors (OXTRs) in the uterus results in initiation of parturition [as reviewed by Kamel (2010)] and contributes to initiation of luteolytic events in some species (Lee et al., 2010). The effects of OT signaling via uterine OXTRs are especially well-characterized in ruminant species. The luteal steroid progesterone (P) can block OXTR signaling via several mechanisms, however, the exact mediator(s) of this response remain to be conclusively identified in uterine tissues.

## Oxytocin and oxytocin receptor (OXTR)

**Figure 1:**
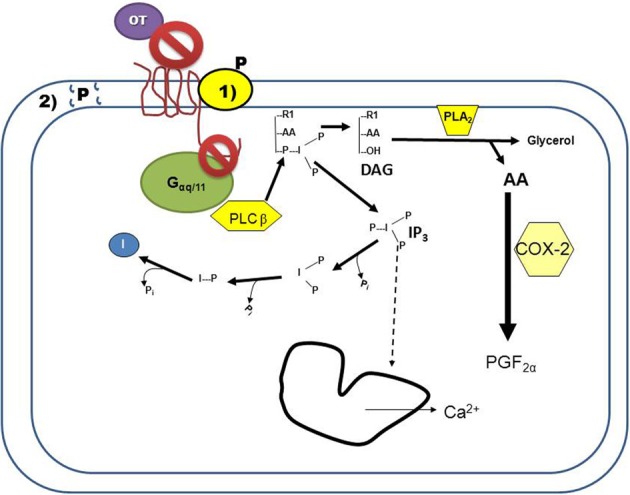
**Progesterone (P) inhibition of oxytocin receptor (OXTR) signaling in endometrial cells.** Binding of oxytocin (OT) to the OXTR activates the G-protein, G_αq/11_. The activated G-protein phosphorylates the enzyme phospholipase Cβ (PLCβ), which cleaves phosphatidylinositol 4,5-bisphosphate into DAG and inositol 1,4,5-trisphosphate (IP_3_). Released IP_3_ induces intracellular calcium release (Ca^2+^) from the endoplasmic reticulum, and is then recycled into free inositol. Free arachadonic acid (AA) is cleaved from DAG by phospholipase A2 (PLA2) and then converted by cyclooxygenase 2 (COX2) into prostaglandin F_2α_ (PGF_2α_). Blockage of this signaling cascade by P can occur by two mechanisms: (1) P binding to a membrane-associated binding protein that interacts with the OXTR, resulting in conformational changes to the receptor such that OT is not able to bind and/or the OXTR is unable to interact with the G-protein. (2) P overloading of the plasma membrane results in changes to membrane fluidity, preventing the OXTR from interacting with the G-protein. Both of these mechanisms of action would result in P-mediated decreased signaling of the OXTR.

OT is a neuropeptide with the amino acid sequence of Cys-Tyr-Ile-Gln-Asn-Cys-Pro-Leu-Gly-NH_2_; OT is secreted primarily from the posterior pituitary, but also by the corpus luteum (CL) of several mammalian species such as swine, ruminants, and primates [as reviewed by Stormshak (2003)]. The OXTR is expressed in OT-target tissues (such as the uterus) and is a class 1 G-protein coupled receptor (GPCR) which primarily activates the G-protein αq/11 upon OT binding [as reviewed by Sanborn et al. (1995)]. Activation of the receptor by Gαq stimulates the enzyme phospholipase (PLC)β, which hydrolyzes phosphatidylinositol 4,5-bisphosphate, generating 1,2 diacylglycerol (DAG) and inositol 1,4,5-trisphosphate (IP_3_). Production of IP_3_ by the OT-stimulated uterine cell causes the endoplasmic reticulum to release stored calcium (Ca^2+^; Figure 1). Intracellular Ca^2+^ release activates protein kinase C (PKC), which phosphorylates many regulatory proteins (Blanks et al., 2007), while free arachidonic acid is cleaved from DAG and converted into prostaglandin F_2α_ (PGF_2α_) in endometrial cells [as reviewed by Gimpl and Fahrenholz (2001)].

### Oxytocin-mediated luteolysis in ruminant species

Functional OXTRs are expressed in the endometrium of the uterus in ruminant species such as the ewe near the end of diestrus (Stevenson et al., 1994). At this time, OT secreted by large luteal cells of the CL (Fields and Fields, 1986) activates OXTR signaling in the endometrium resulting in secretion of PGF_2α_ [as reviewed by Niswender et al. (2007)]. Endometrial PGF_2α_ is transported directly via ovarian artery to the CL where it induces further release of OT (Lee et al., 2010), in a positive feedback loop that ultimately results in luteal regression. The ruminant conceptus secretes interferon tau (IFNT), which acts to block/prevent OXTR signaling and prolong luteal life span [as reviewed by Spencer et al. (2007)].

The involvement of OT and OXTR in luteal regression of primates is still controversial. Unlike ruminant CL, transplantation of the ovary away from the uterine blood supply does not prolong luteal lifespan in primates, indicating an intraluteal mechanism of luteolysis (Burford and Diddle, 1936; Beavis et al., 1969). Several studies in non-human primates suggest that OT increases P secretion from early luteal phase CL, and might be involved with maintenance of gap-junctions in luteal tissue (Khan-Dawood et al., 1998). More recently, expression of functional OXTR was reported in human luteinized granulosa cells (Saller et al., 2010). Activation of the OXTR by OT increases caspase signaling and induces morphological changes to luteinized granulosa cells associated with regression. Therefore, while there is evidence that the OXTR is involved in primate luteal regression, additional well-designed studies are needed to precisely define its role in primate luteolysis.

## Effects of progesterone on OXTR gene expression

Following ovulation and luteinization in the ewe, during diestrus there is an initial increase in expression of the genomic P receptor (PGR) in the uterine endometrial glands, and a concurrent absence of OXTRs. Blockage of endometrial OXTR expression by ligand-bound PGR is via an indirect mechanism resulting from P suppression of estrogen receptors (ERs) [as reviewed by Spencer and Bazer (2004)]. However, even when OXTR are expressed in endometrial cells, exposure of the endometrium to both estradiol (E) and P is absolutely necessary for OT to induce PGF_2α_ secretion (Vallet et al., 1990). This is most likely due to P modulation and/or up-regulation of the necessary precursors in endometrial cells for prostaglandin production [as reviewed by Schams and Berisha (2002)]. The ruminant OXTR has been cloned; the promoter region contains several AP-1 and SP1 transcription factor recognition sites, as well as PGR half-sites (Fleming et al., 2006); genomic regulation of OXTR is mediated by ERα (ESR1) via these SP-1 sites, with no direct regulation by PGR. Similarly, IFNT also decreases ESR1 expression, thereby preventing ESR1-induced transcription of the OXTR gene (Fleming et al., 2006).

Increased expression of OXTR near parturition is noted in all mammalian species, including primates and ruminants; OXTR signaling is critical for initiation of myometrial contractions in ruminants (Wathes and Hamon, 1993). Genomic PGR is expressed in near-term myometrium, but PGR-coactivators decline prior to initiation of labor in women and mice (Condon et al., 2003). Administration of mifepristone (PGR antagonist) to women for blockage of PGR signaling can initiate labor [Cochrane review Hapangama and Neilson (2009)]. This suggests direct suppression of the OXTR near term by P via PGR signaling. But, regulation of OXTR gene function by P in pregnant myometrium is most likely indirect via immunosuppressive actions of P [as reviewed by Arck et al. (2007)]. Recently, binding sites for nuclear factor-kappa B p65 (RELA) and CCAAT/enhancer-binding protein (CEBP) were identified in the human OXTR gene promotor region (Khanjani et al., 2011). Expression of CEBP and RELA are increased in inflammatory responses (Adams et al., 2000), and mediators of both are increased in near-term myometrium of women (Khanjani et al., 2011).

Most notably, microarray analyses of P-response genes in murine endometrium at early pregnancy did not find that the *Oxtr* gene was altered by progestin replacement following ovariectomy (Jeong et al., 2005). Moreover, while mRNA for OXTR is expressed in rhesus luteal tissue during the mid-late luteal phase (identified by microarray analysis), it is not regulated by progestin [GEO dataset Series GSE12281 (Bishop et al., 2009)]. Given these data along with the previously noted studies in ruminants and women, it is likely that P regulation of the OXTR gene is indirect via modulation of other factors, such as ESR1 and immune factors.

## Non-genomic inhibition of OXTR signaling by P; evidence of biological significance

Evidence for non-genomic inhibition of uterine OXTR signaling includes the observation that P and metabolites of P rapidly inhibit contractions of rat uteri within 2 min of treatment; this response is not opposed by treatment with the PGR antagonist RU 486 (Putnam et al., 1991). While these authors suggest that GABA_A_ receptors mediate these effects, the rodent uterus also secretes OT, suggesting that the effects of P might be via non-genomic inhibition of rodent *Oxtr* signaling. Indeed, later studies from Grazzini et al. (1998) demonstrated that P induces a reduction in rat uterine OT signaling without a change in *Oxtr* mRNA expression.

Later studies in non-pregnant ruminants (Bogacki et al., 2002) demonstrated that P could inhibit OT-induced intracellular calcium release and PGF_2α_ secretion by bovine endometrial tissue explants in the presence of actinomycin D, an inhibitor of DNA transcription/mRNA synthesis. However, subsequent experiments revealed P as well as pregnenolone, 17β-hydroxyprogesterone, the PR antagonist onapristone, and testosterone at micromolar (μM) concentrations were all able to interfere with OT-stimulated PGF_2α_ secretion and intracellular calcium release (Duras et al., 2005). Inhibition of both OT-induced IP_3_ accumulation and PGF_2α_ release from ovine endometrial explants was later observed at lower nM concentrations of P within hours (Bishop and Stormshak, 2006). This rapid, specific inhibition of OXTR signaling by P was confirmed in COS7 (African green monkey kidney) cells transfected with the ovine OXTR that lack PGRs (Bishop et al., 2008).

Incubation of myometrial strips from women at term pregnancy with P decreased OT-induced contractility within 20 min to an hour in a concentration–dependent manner, with maximal inhibition at 10 μM P (Chanrachakul et al., 2005). This could indicate a role for non-genomic inhibition of OXTR signaling by P to prevent premature onset of parturition.

### Molecular actions of non-genomic inhibition of OXTR signaling by P

Rapid inhibition of rodent *Oxtr* signaling is due to a P-mediated decrease in OT binding capacity, not binding affinity (Grazzini et al., 1998). Inhibition of OT binding is P dose-dependent and specific to the *Oxtr* (i.e., no effect on a related GPCR, vasopressin receptor). The observed inhibition by P of *Oxtr* function is at the level of the plasma membrane: P conjugated to bovine serum albumin, which prevents P from crossing through the plasma membrane easily, also inhibited membrane binding of OT. Specific and high-affinity binding sites for P were reported only in transfected CHO cells expressing the *Oxtr*. Moreover, high-affinity binding of P to *Oxtr*-expressing membranes was regulated by the state of the G-protein coupling to the receptor, based on additional experiments performed with the G-protein uncoupling reagent GTPγ S. These observations indicate that the interaction of P with the rodent *Oxtr* is possibly a direct steroid-receptor interaction, either by altering the conformation of the receptor or directly blocking the binding site for OT on the OXTR (Grazzini et al., 1998).

Later investigations of the ovine OXTR validated these initial studies in rodents. Isolated membrane fractions from the uterine luminal epithelium of artificially-cycled, ovariectomized ewes pre-incubated with P showed decreased OT binding, which was reversed by co-incubation with RU 486 (Dunlap and Stormshak, 2004). The presence of high-affinity membrane binding sites for P were identified, with P saturation occurring at 8 nM (K_d_= 1.2 × 10^−9^ M). E, cortisol, testosterone, and arginine vasopressin failed to compete for the P binding site, however, P, R5020 (synthetic progestin), RU 486, and OT all bound to the same site on plasma membranes. A radioreceptor exchange assay demonstrated that pre-exposure of membranes to P significantly increased the number of P binding sites. Inhibition of OT binding to endometrial membranes by P was dose-dependent at a low (nM) steroid concentration (Bishop and Stormshak, 2006). However, unlike the rodent *Oxtr* (Grazzini et al., 1998) and native OXTR expression in ovine endometrial membranes (Dunlap and Stormshak, 2004), P does not decrease specific OT binding to the ovine OXTR when transfected into the COS7 (African green monkey kidney fibroblast) cell line (Bishop et al., 2008). Additionally, no specific P binding sites were identified in membranes of OXTR-transfected COS7 cells. This cell line (COS7) was chosen for transfection due to the fact that it lacked P receptors. While CHO (Chinese hamster ovary) cells are not reported to contain PGRs, the observed discrepancy between the two experiments (Grazzini et al., 1998; Bishop et al., 2008) could be due to the fact that CHO cells are of reproductive tract origin (unlike COS7) and may express a P binding protein.

Effects of progestins to inhibit a primate OXTR transfected into various cell lines was only investigated using μM concentration of steroid (Burger et al., 1999). This study reported that P inhibited the signaling of several related GPCRs (including acetylcholine receptor and vasopressin receptor) as well as the OXTR, suggesting that these effects are non-specific at μM concentrations of P.

### Possible non-genomic mediators of P-inhibition of OXTR signaling

As noted in several studies (Burger et al., 1999; Duras et al., 2005), μM concentrations of many classes of steroids have non-specific effects on OXTR signaling apparently in absence of specific binding of steroid to a single receptor. Non-receptor-mediated effects of various steroids, including P, were reported from studies of lipid domains using artificial membrane bilayers (Wenz and Barrantes, 2003). The lower the hydrophobicity of the steroid, (determined by the group bound to C17) the more disruptive the steroid is to lipid domains at μM molar concentrations. The composition and presence of these lipid domains in cell membranes may be critical to GPCR signaling (Bruno et al., 2012). P, promegestone, pregnenolone, 11 α-hydroxyprogesterone, and 17 α- hydroxypregnenolone all possess low hydrophobicity groups attached to C17 and demonstrate domain-disrupting activity at μM concentrations (Wenz and Barrantes, 2003). The previously mentioned study of Burger et al. (1999), found that the mechanism by which P inhibited signaling of several related GPCRs was possibly due to an observed decrease in plasma membrane fluidity. Therefore, the non-specific effect of several steroids on OXTR signaling at higher μM concentrations may be due to disruption of the lipid bilayer by steroid overloading of the plasma membrane. Steroid overloading of the membranes may affect OXTR coupling to the G-protein and activation of downstream signaling effectors by changing the fluidity of the plasma membrane (Gimpl et al., 2002), and preventing conformational changes of the receptor from inducing downstream effectors. This is a likely mechanism when local levels of steroids are high, such as during gestation (from placental P) and in the luteal tissue itself. In bovine CL, OXTRs are localized to pure preparations of small, steroidogenic luteal cells (SLCs), which release Ca^2+^ in response to OT-stimulus (Davis et al., 2010). If these SLCs are pre-incubated for 1 h with a high concentration of P (~100 μM), the OT-stimulated intracellular Ca^2+^ release is blocked. Because this phenomena was mimicked by replacing P with a cyclodextrin (methyl-β cyclodextrin), P might be acting to exclude cholesterol from the plasma membrane, thus altering membrane fluidity in these SLCs (Davis et al., 2010).

However, data generated from experiments in ovine endometrial tissue (Dunlap and Stormshak, 2004; Bishop and Stormshak, 2006), demonstrating a specific and saturable binding site for P that is closely associated with the OXTR suggests that rapid inhibition of OXTR signaling by P in reproductive tissues may be receptor mediated. A non-PGR, P receptor membrane component 1 (PGRMC1) and associated serpine mRNA binding protein 1 (SERBP1) are expressed in the uterus (endometrium and myometrium) of ruminants (Luciano et al., 2011) and non-human primates (Keator et al., 2012), and in human ovarian tissue (Luciano et al., 2011). Expression and actions of these proteins are extensively characterized in granulosa cells (Peluso et al., 2006), where the main function is preventing apoptosis. Given the fact that OXTR is expressed in primate luteal tissue and OT might contribute to intra-luteal initiation/progression of primate luteolysis (Saller et al., 2010); a membrane PR may prevent local OXTR signaling in the CL. Our research group observed expression of PGRMC1 protein present in the granulosa lutein cells of the rhesus macaque CL throughout the entire luteal phase, with intense staining noted in distinct subpopulations of cells clustered around the periphery of the CL (Bishop, Bogan, Slayden, Hennebold, and Stouffer, Unpublished). Expression of PGRMC1 mRNA in luteal tissue peaks at the late luteal phase, just prior the fall in P levels, and PGRMC1 protein is increased following chorionic gonadotropin exposure simulating early pregnancy (Bishop et al., 2011). In the ruminant uterus, evaluation of PGRMC1 expression by immunohistochemistry showed robust staining of bovine luminal epithelium (Luciano et al., 2011), the same cell type express OXTR (Leung et al., 2001). Possible effects and/or interactions between PGRMC1 and OXTR in the plasma membrane of both the endometrium and the CL remain to be investigated.

Other novel, non-genomic membrane PRs (mPRs) have been identified in the ovine uterus which may act to block local OXTR signaling (Ashley et al., 2009). While the function(s) of the mPR have yet to be described in the ewe, it reportedly stimulates intracellular calcium release. Three homologues of the mPR are present in the human uterus: mPRα, β, and γ (Fernandes et al., 2005). Endometrial mRNA expression of mPRα and mPRβ differ by the stage of the menstrual cycle; both decrease at the onset of parturition (Fernandes et al., 2005). These mPR are GPCRs, functioning to inhibit adenylcyclase activity (coupled to Gα_i_). The effect these mPRs might exert on OXTR signaling in uterine tissues remains to be investigated.

The genomic PGR also rapidly activates the MAPK pathway upon stimulation in breast cancer cells via a SRC kinase-like SH3 domain (Faivre et al., 2005; Skildum et al., 2005). The OXTR can also activate the MAPK pathway (Devost et al., 2008), but given the evidence that interference of OT binding to the OXTR occurs at the level of the plasma membrane (Grazzini et al., 1998; Dunlap and Stormshak, 2004; Bishop and Stormshak, 2006), involvement of the cytoplasmic/nuclear PGR to directly block OXTR signaling is unlikely. There is limited data on membrane-associated PGR, however, a microarray-based analysis did identify a membrane-associated mRNA encoding the PGR, suggesting localization to the plasma membrane (Diehn et al., 2006). Further studies are needed to clarify any non-genomic effects of PGR to block OXTR signaling.

## Conclusions

There is evidence that P directly antagonizes OXTR signaling via a non-genomic mechanism, however, there are only a few studies, some with conflicting results (summarized in Table 1). Experiments conducted on the ruminant OXTR demonstrate rapid blockage of OT-stimulated secretion of PGF_2α_ from endometrial tissue by P, which could lead to delayed luteolytic events. The best-characterized tissue to date of regulation of OXTR expression and OT signaling is the ruminant uterus, however, rapid inhibition of OT action by P remains to be investigated in the ruminant uterus during pregnancy. Inhibition of OXTR signaling may be due to non-receptor mediated events related to steroid overloading of plasma membranes, or via specific binding of P to one of several membrane-associated P receptors (Figure 1). Nevertheless, while there is ample evidence supporting an OXTR-associated P binding site in *situ*, further studies are needed to elucidate any involvement of membrane P receptors in blockage of OXTR signaling. The role of an ovarian OXTR in regulation of primate luteal tissue remains unclear, but P may act to block local OXTR signaling. Given recent studies identifying functional OXTR in primate luteal cells and P inhibition of OT-signaling in bovine SLCs (Davis et al., 2010), further experiments could help identify causes of fertility disorders associated with luteal function in women. Additionally, molecular mechanisms regulating parturition and onset of labor in women are still being defined. Greater understanding of OXTR signaling in the primate uterus might help reduce complications from onset of preterm labor (Hubinont and Debieve, 2011).

**Table 1:** **Studies of progesterone inhibition of OXTR signaling**.

**Species**	**Citation**	**Major findings**
Rodent	Putnam et al., 1991	P and P metabolites rapidly inhibit contractions of rat uteri within 2 min of treatment
		Effect of P not opposed by treatment with the PGR antagonist RU 486
	Grazzini et al., 1998	P reduced rat uterine OT signaling without a change in *Oxtr* mRNA expression
		Inhibition due to a P-mediated decrease in OT binding capacity, not binding affinity
		Occurred at the level of the plasma membrane
		Specific and high affinity binding sites for P only in transfected CHO cells expressing *Oxtr* with high affinity
		Regulated by the state of the G-protein coupling to the receptor
Ovine	Dunlap and Stormshak, 2004	Isolated membrane fractions pre-incubated with P showed decreased OT binding, reversed by co-incubation with RU 486
		High affinity binding site for P
		P, R5020 (synthetic progestin), RU 486, and OT all bind to the same site pre-exposure of membranes to P significantly increased the number of P binding sites
	Bishop and Stormshak, 2006	P binding to endometrial membranes is dose dependent
		Inhibition of both OT-induced IP3 accumulation and PGF_2α_ release from ovine endometrial explants by P within hours of exposure
	Bishop et al., 2008	Rapid, specific inhibition of OXTR signaling by P in COS7 cells transfected with the ovine OXTR that lack PGRs
		P did not decrease specific binding of OT
		No specific P binding sites in membranes of OXTR-transfected cells
Bovine	Bogacki et al., 2002	P inhibited OT binding at all concentrations (20–0.002 μM) investigated
		OT-induced intracellular calcium release and PGF_2α_ secretion by bovine endometrial tissue explants in the presence of actinomycin D
	Duras et al., 2005	P as well as pregnenolone, 17β-hydroxyprogesterone, the PR antagonist onapristone, and testosterone at μM concentrations were all able to interfere with OT-stimulated PGF_2α_ secretion and intracellular calcium release
	Davis et al., 2010	P at μM concentration rapidly (within 1 h) inhibited OT-induced Ca^2+^ release from endoplasmic reticulum of small steroidogenic luteal cells, possibly via exclusion of cholesterol from the luteal cell plasma membrane
Primate/Human	Burger et al., 1999	μM concentration of P inhibited the signaling of several related GPCRs as well as the OXTR
	Chanrachakul et al., 2005	Within 20 min-1 h P decreased OT-induced contractility of term myometrium in a concentration dependent manner
		Maximal inhibition at 10 μM P

### Conflict of interest statement

The author declares that the research was conducted in the absence of any commercial or financial relationships that could be construed as a potential conflict of interest.

## References

[B1] Adams D. S. Nathans R. Pero S. C. Sen A. Wakshull E. (2000). Activation of a rel-A/CEBP-beta-related transcription factor heteromer by PGG-glucan in a murine monocytic cell line. J. Cell Biochem. 77, 221–233 10723089 10.1002/(sici)1097-4644(20000501)77:2<221::aid-jcb6>3.0.co;2-v

[B2] Arck P. Hansen P. J. Mulac Jericevic B. Piccinni M. P. Szekeres-Bartho J. (2007). Progesterone during pregnancy: endocrine-immune cross talk in mammalian species and the role of stress. Am. J. Reprod. Immunol. 58, 268–279 10.1111/j.1600-0897.2007.00512.x 17681043

[B3] Ashley R. L. Arreguin-Arevalo J. A. Nett T. M. (2009). Binding characteristics of the ovine membrane progesterone receptor alpha and expression of the receptor during the estrus cycle. Reprod. Biol. Endocrinol. 7, 42 19432978 10.1186/1477-7827-7-42 PMC2685384

[B4] Beavis E. L. Brown J. B. Smith M. A. (1969). Ovarian function after hysterectomy with conservation of the ovaries in pre-menopausal women. Int. J. Obstet. Gynaecol. 76, 969–978 5355381 10.1111/j.1471-0528.1969.tb09462.x

[B5] Bishop C. V. Filtz T. Zhang Y. Slayden O. Stormshak F. (2008). Progesterone suppresses an oxytocin-stimulated signal pathway in COS-7 cells transfected with the oxytocin receptor. Steroids 73, 1367–1374 10.1016/j.steroids.2008.06.014 18674552 PMC2630247

[B6] Bishop C. V. Hennebold J. D. Stouffer R. L. (2009). The effects of luteinizing hormone ablation/replacement versus steroid ablation/replacement on gene expression in the primate corpus luteum. Mol. Hum. Reprod. 15, 181–193 10.1093/molehr/gap005 19168862 PMC2647108

[B7] Bishop C. V. Satterwhite S. Xu L. Hennebold J. D. Stouffer R. L. (2011). Microarray analysis of the primate luteal transcriptome during chorionic gonadotrophin administration simulating early pregnancy. Mol. Hum. Reprod. 18, 216–227 10.1093/molehr/gar073 22072816 PMC3350325

[B8] Bishop C. V. Stormshak F. (2006). Nongenomic action of progesterone inhibits oxytocin-induced phosphoinositide hydrolysis and prostaglandin F2alpha secretion in the ovine endometrium. Endocrinology 147, 937–942 10.1210/en.2005-0869 16254031

[B9] Blanks A. M. Shmygol A. Thornton S. (2007). Regulation of oxytocin receptors and oxytocin receptor signaling. Semin. Reprod. Med. 25, 52–59 10.1055/s-2006-956775 17205423

[B10] Bogacki M. Silvia W. J. Rekawiecki R. Kotwica J. (2002). Direct inhibitory effect of progesterone on oxytocin-induced secretion of prostaglandin F_2*a*_ from bovine endometrial tissue. Biol. Reprod. 67, 184–188 10.1095/biolreprod67.1.184 12080016

[B11] Bruno A. Costantino G. De Fabritiis G. Pastor M. Selent J. (2012). Membrane-sensitive conformational states of helix 8 in the metabotropic Glu2 receptor, a class C GPCR. PLoS ONE 7:e42023 10.1371/journal.pone.0042023 22870276 PMC3411606

[B12] Burford T. H. Diddle A. W. (1936). Effect of total hysterectomy upon the ovary of the Macacus rhesus monkey. Surg. Gynecol. Obstet. 62, 600–609

[B13] Burger K. Fahrenholz F. Gimpl G. (1999). Non-genomic effects of progesterone on the signaling function of G protein-coupled receptors. FEBS Lett. 464, 25–29 10.1016/S0014-5793(99)01668-3 10611477

[B14] Chanrachakul B. Broughton Pipkin F. Warren A. Y. Arulkumaran S. Khan R. N. (2005). Progesterone enhances the tocolytic effect of ritodrine in isolated pregnant human myometrium. Am. J. Obstet. Gynecol. 192, 458–463 10.1016/j.ajog.2004.07.077 15695987

[B15] Condon J. C. Jeyasuria P. Faust J. M. Wilson J. W. Mendelson C. R. (2003). A decline in the levels of progesterone receptor coactivators in the pregnant uterus at term may antagonize progesterone receptor function and contribute to the initiation of parturition. Proc. Natl. Acad. Sci. U.S.A. 100, 9518–9523 10.1073/pnas.1633616100 12886011 PMC170950

[B16] Davis T. L. Bott R. C. Slough T. L. Bruemmer J. E. Niswender G. D. (2010). Progesterone inhibits oxytocin- and prostaglandin F2alpha-stimulated increases in intracellular calcium concentrations in small and large ovine luteal cells. Biol. Reprod. 82, 282–288 10.1095/biolreprod.109.079970 19812299 PMC2809223

[B17] Devost D. Wrzal P. Zingg H. H. (2008). Oxytocin receptor signaling. Prog. Brain Res. 170, 167–176 10.1016/S0079-6123(08)00415-9 18655881

[B18] Diehn M. Bhattacharya R. Botstein D. Brown P. O. (2006). Genome-scale identification of membrane-associated human mRNAs. PLoS Genet 2:e11 10.1371/journal.pgen.0020011 16415983 PMC1326219

[B19] Dunlap K. A. Stormshak F. (2004). Nongenomic inhibition of oxytocin binding by progesterone in the ovine uterus. Biol. Reprod. 70, 65–69 10.1095/biolreprod.103.020180 12954727

[B20] Duras M. Mlynarczuk J. Kotwica J. (2005). Non-genomic effect of steroids on oxytocin-stimulated intracellular mobilization of calcium and on prostaglandin F2alpha and E2 secretion from bovine endometrial cells. Prostaglandins Other Lipid. Mediat. 76, 105–116 10.1016/j.prostaglandins.2005.02.001 15967166

[B21] Faivre E. Skildum A. Pierson-Mullany L. Lange C. A. (2005). Integration of progesterone receptor mediated rapid signaling and nuclear actions in breast cancer cell models: role of mitogen-activated protein kinases and cell cycle regulators. Steroids 70, 418–426 10.1016/j.steroids.2005.02.012 15862825

[B22] Fernandes M. S. Pierron V. Michalovich D. Astle S. Thornton S. Peltoketo H. (2005). Regulated expression of putative membrane progestin receptor homologues in human endometrium and gestational tissues. J. Endocrinol. 187, 89–101 10.1677/joe.1.06242 16214944

[B23] Fields M. J. Fields P. A. (1986). Luteal neurophysin in the nonpregnant cow and ewe: immunocytochemical localization in membrane-bounded secretory granules of the large luteal cell. Endocrinology 118, 1723–1725 10.1210/endo-118-4-1723 3948797

[B24] Fleming J. G. Spencer T. E. Safe S. H. Bazer F. W. (2006). Estrogen regulates transcription of the ovine oxytocin receptor gene through GC-rich SP1 promoter elements. Endocrinology 147, 899–911 10.1210/en.2005-1120 16254027

[B25] Gimpl G. Fahrenholz F. (2001). The oxytocin receptor system: structure, function, and regulation. Physiol. Rev. 81, 629–683 11274341 10.1152/physrev.2001.81.2.629

[B26] Gimpl G. Wiegand V. Burger K. Fahrenholz F. (2002). Cholesterol and steroid hormones: modulators of oxytocin receptor function. Prog Brain Res 139, 43–55 10.1016/S0079-6123(02)39006-X 12436925

[B27] Grazzini E. Guillon G. Mouillac B. Zingg H. H. (1998). Inhibition of oxytocin receptor function by direct binding of progesterone. Nature 392, 509–512 10.1038/33176 9548257

[B28] Hapangama D. Neilson J. P. (2009). Mifepristone for induction of labour. Cochrane Database Syst. Rev. 3:CD002865 10.1002/14651858.CD002865.pub2 19588336 PMC3992376

[B29] Hubinont C. Debieve F. (2011). Prevention of preterm labour: 2011 update on tocolysis. J. Pregnancy. 2011, 941057 22175022 10.1155/2011/941057 PMC3228310

[B30] Jeong J. W. Lee K. Y. Kwak I. White L. D. Hilsenbeck S. G. Lydon J. P. (2005). Identification of murine uterine genes regulated in a ligand-dependent manner by the progesterone receptor. Endocrinology 146, 3490–3505 10.1210/en.2005-0016 15845616

[B31] Kamel R. M. (2010). The onset of human parturition. Arch. Gynecol. Obstet. 281, 975–982 10.1007/s00404-010-1365-9 20127346

[B32] Keator C. S. Mah K. Slayden O. D. (2012). Alterations in progesterone receptor membrane component 2 (PGRMC2) in the endometrium of macaques afflicted with advanced endometriosis. Mol. Hum. Reprod. 18, 308–319 10.1093/molehr/gas006 22307145 PMC3358041

[B33] Khan-Dawood F. S. Yang J. Dawood M. Y. (1998). Hormonal regulation of connexin-43 in baboon corpora lutea. J. Endocrinol. 157, 405–414 10.1677/joe.0.1570405 9691973

[B34] Khanjani S. Terzidou V. Lee Y. S. Thornton S. Johnson M. R. Bennett P. R. (2011). Synergistic regulation of human oxytocin receptor promoter by CCAAT/enhancer-binding protein and RELA. Biol. Reprod. 85, 1083–1088 10.1095/biolreprod.111.092304 21734268

[B35] Lee J. Mc Cracken J. A. Banu S. K. Rodriguez R. Nithy T. K. Arosh J. A. (2010). Transport of prostaglandin F(2alpha) pulses from the uterus to the ovary at the time of luteolysis in ruminants is regulated by prostaglandin transporter-mediated mechanisms. Endocrinology 151, 3326–3335 10.1210/en.2009-0948 20410207

[B36] Leung S. T. Cheng Z. Sheldrick E. L. Derecka K. Flint A. P. F. Wathes D. C. (2001). The effects of lipopolysaccharide and interleukins-1alpha, -2 and -6 on oxytocin receptor expression and prostaglandin production in bovine endometrium. J. Endocrinol. 168, 497–508 10.1677/joe.0.1680497 11241181

[B37] Luciano A. M. Corbani D. Tessaro I. Franciosi F. Peluso J. J. Modina S. (2011). Expression of progesterone membrane component-1 in bovine reproductive system druing estrus cycle. Eur. J. Histochem. 55:e27 10.4081/ejh.2011.e27 22073374 PMC3203473

[B38] Niswender G. D. Davis T. L. Griffith R. J. Bogan R. L. Monser K. Bott R. C. (2007). Judge, jury and executioner: the auto-regulation of luteal function. Soc. Reprod. Fertil. Suppl. 64, 191–206 17491148 10.5661/rdr-vi-191

[B39] Peluso J. J. Pappalardo A. Losel R. Wehling M. (2006). Progesterone membrane receptor component 1 expression in the immature rat ovary and its role in mediating progesterone's antiapoptotic action. Endocrinology 147, 3133–3140 10.1210/en.2006-0114 16513825

[B40] Putnam C. Brann D. Kolbeck R. Mahesh V. (1991). Inhibition of uterine contractility by progesterone and progesterone metabolites: mediation by progesterone and gamma-aminobutyric acid_A_ receptor systems. Biol. Reprod. 45, 266–272 10.1095/biolreprod45.2.266 1664743

[B41] Saller S. Kunz L. Dissen G. A. Stouffer R. Ojeda S. R. Berg D. (2010). Oxytocin receptors in the primate ovary: molecular identity and link to apoptosis in human granulosa cells. Hum. Reprod. 25, 969–976 10.1093/humrep/dep467 20097922 PMC2839908

[B42] Sanborn B. M. Qian A. Ku C. Y. Wen Y. Anwer K. Monga M. (1995). Mechanisms regulating oxytocin receptor coupling to phospholipase C in rat and human myometrium. Adv. Exp. Med. Biol. 395, 469–479 8713999

[B43] Schams D. Berisha B. (2002). Steroids as local regulators of ovarian activity in domestic animals. Domest. Anim. Endocrinol. 23, 53–65 10.1016/S0739-7240(02)00145-5 12142226

[B44] Skildum A. Faivre E. Lange C. A. (2005). Progesterone receptors induce cell cycle progression via activation of mitogen-activated protein kinases. Mol. Endocrinol. 19, 327–339 10.1210/me.2004-0306 15486045

[B45] Spencer T. E. Bazer F. W. (2004). Conceptus signals for establishment and maintenance of pregnancy. Reprod. Biol. Endocrinol. 2, 49 15236653 10.1186/1477-7827-2-49 PMC471568

[B46] Spencer T. E. Johnson G. A. Bazer F. W. Burghardt R. C. (2007). Fetal-maternal interactions during the establishment of pregnancy in ruminants. Soc. Reprod. Fertil. Suppl. 64, 379–396 17491160 10.5661/rdr-vi-379

[B47] Stevenson K. R. Riley P. R. Stewart H. J. Flint A. P. F. Wathes D. C. (1994). Localization of oxytocin receptor mRNA in the ovine uterus during the oestrous cycle and early pregnancy. J. Mol. Endocrinol. 12, 93–105 10.1677/jme.0.0120093 8185818

[B48] Stormshak F. (2003). Biochemical and endocrine aspects of oxytocin production by the mammalian corpus luteum. Reprod. Biol. Endocrinol. 1, 92 14613532 10.1186/1477-7827-1-92 PMC280731

[B49] Vallet J. L. Lamming G. E. Batten M. (1990). Control of endometrial oxytocin receptor and uterine response to oxytocin by progesterone and oestradiol in the ewe. J. Reprod. Fertil. 90, 625–634 10.1530/jrf.0.0900625 2174461

[B50] Wathes D. C. Hamon M. (1993). Localization of oestradiol, progesterone and oxytocin receptors in the uterus during the oestrous cycle and early pregnancy of the ewe. J. Endocrinol. 138, 479–492 10.1677/joe.0.1380479 8277221

[B51] Wenz J. J. Barrantes F. J. (2003). Steroid structural requirements for stabilizing or disrupting lipid domains. Biochemistry 42, 14267–14276 14640695 10.1021/bi035759c

